# Evaluation of Strain Values for Masseter Muscle Activity of Dentofacial Deformities Using Ultrasound Elastography

**DOI:** 10.3390/jcm14217769

**Published:** 2025-11-01

**Authors:** Yutaka Sasajima, Kazuhiro Ooi, Takako Terakami, Rei Jokaji, Hirokazu Okita, Yusuke Nakade, Shuichi Kawashiri

**Affiliations:** 1 Department of Oral and Maxillofacial Surgery, Kanazawa University Graduate School of Medical Science, Kanazawa 920-8641, Japan; sasajima29346@gmail.com (Y.S.); r_johkaji@staff.kanazawa-u.ac.jp (R.J.); skawa@med.kanazawa-u.ac.jp (S.K.); 2 Department of Clinical Laboratory, Kanazawa University Hospital, Kanazawa 920-8641, Japan; terakami@staff.kanazawa-u.ac.jp (T.T.); nakadeyukeitu@gmail.com (Y.N.); 3 Department of Rehabilitation Medicine, Kanazawa University Hospital, Kanazawa 920-8641, Japan; okita@med.kanazawa-u.ac.jp

**Keywords:** ultrasound elastography, strain ratio, dentofacial deformities, masseter muscle activity

## Abstract

**Background/Objective**: This study aimed to evaluate the strain values (SVs) of masseter muscle activity in dentofacial deformities (DDs) using ultrasound elastography. **Methods**: The DD group consisted of 60 patients with dentofacial deformities with skeletal class II or III malocclusion, and the control group consisted of 26 volunteers with normal occlusion. The SVs and the cross-sectional area of the masseter muscle were measured using an ultrasonic elastography. These were measured at three functional positions: resting, mouth opening, and clenching. The SVs were statistically compared with the DD and control groups. Changes in the cross-sectional area and factors (patient status, skeletal morphology, and oral function) related to the SVs in the study group were statistically analyzed. **Results**: The SVs were significantly higher during clenching than in resting and mouth opening in both groups, although there was no correlation between the DD group and the control group. The cross-sectional area was larger during clenching, resting, and mouth opening. The resting SVs decreased as the masseter muscle cross-sectional area increased. The SVs of clenching increased with higher resting SVs and decreased with greater maximum mouth opening. **Conclusions**: The SVs measured by ultrasound elastography changed depending on functional mandibular movement and have the potential to evaluate the masseter muscle activity of dentofacial deformities.

## 1. Introduction

Dentofacial deformities are accompanied by abnormalities in dentofacial morphology and functional disorders due to abnormalities in the maxillo-mandibular skeletal morphology, position, and intermaxillary relationship between the maxilla and mandible. Evaluation of masticatory muscles is considered important in jaw movements, such as mouth opening and mastication. Functional evaluation is required, especially in dentofacial deformities where masticatory disorders are observed. Methods for evaluating masticatory muscles in dentofacial deformities include computed tomography (CT) and magnetic resonance imaging (MRI) for morphological evaluation and electromyography for functional evaluation [1,2,3]. However, owing to radiation exposure and the complexity of the examination, a new evaluation method that can easily evaluate the function is required.

Ultrasound elastography is an emerging non-invasive imaging modality that provides information on tissue elasticity. Among its techniques, strain elastography allows for the semi-quantitative evaluation of muscle stiffness, which may reflect underlying muscle function and pathology [4,5]. Previous studies have shown the utility of ultrasound elastography in evaluating skeletal muscle injuries and chronic muscle diseases [6,7], but its application in the field of maxillofacial surgery remains limited. The masseter muscle plays a crucial role in jaw movement and masticatory performance. The differences in the masseter muscle between jaw deformities and healthy subjects and the stiffness of the masseter muscle during function have not been reported [8,9].

The relationship between postoperative recurrence and adaptation of masticatory muscles has also been discussed [10], and objective evaluation of masticatory muscles is considered important. If ultrasound elastography can be applied to the evaluation of masticatory muscles, an objective and more accurate diagnosis can be expected, but sufficient knowledge does not currently exist. The purpose of this study was to apply ultrasound elastography to investigate functional changes in the masseter muscle in patients with skeletal class II and III dentofacial deformities and the factors associated with these changes.

## 2. Methods

### 2.1. Ethics

This study complied with the principles stated in the Declaration of Helsinki Ethical Principles for Medical Research Involving Human Subjects, adopted by the 18th World Medical Assembly, Helsinki, Finland, June 1964, and was amended most recently by the 64th World Medical Assembly, Fontaleza, Brazil, October 2013. This study was approved by the Kanazawa University Hospital Research Ethics Committee (Ref. No. 2963-1, date of approval: 20 February 2019).

#### 2.1.1. Patients Informed Consent

All of the patients were informed of the research purpose and agreed to provide their clinical data for this study.

#### 2.1.2. Inclusion Criteria

Patients with skeletal class II and III malocclusion were included. Skeletal class II malocclusion (*n* = 14) was defined as an ANB angle > 4° on lateral cephalometric analysis, whereas skeletal class III malocclusion (*n* = 46) was defined as an ANB angle < 1° or a Wits appraisal < −2 mm. All patients first visited the Department of Oral and Maxillofacial Surgery, Kanazawa University Hospital, between January 2019 and January 2022. The control group consisted of volunteers with normal occlusion (*n* = 26) recruited from hospital staff. All participants were aged 16–59 years and had complete permanent dentition excluding third molars.

#### 2.1.3. Exclusion Criteria

Patients with congenital malformations (including cleft palate), temporomandibular disorders, pain, and trismus were excluded from this study.

### 2.2. Strain Values and Cross-Sectional Area Measurement of the Masseter Muscle by Ultrasound Elastography

**Figure 1:**
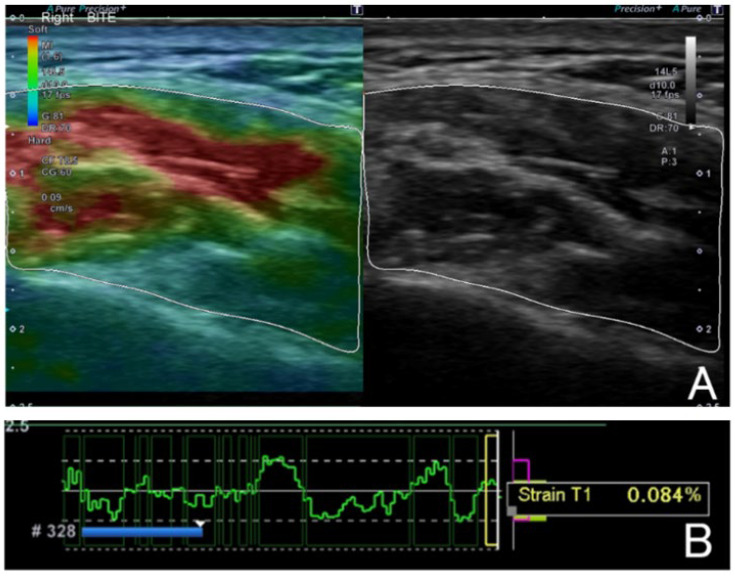
Ultrasound elastography image. Regions of interest (ROI) are indicated by white rectangles and were set to masseter muscle excluding fascia and fat. Color coded from red (high revel strain ratio) to blue (low level strain ratio) according to the hardness of tissue by B-mode images (**A**). There is a scale that shows the rate of compression and relaxation cycles, and the masseter muscle area is selected when it is constant (**B**).

The strain values (SVs) and cross-sectional area were measured using an ultrasonic elastography device (ultrasonic testing device Aplioi700, manufactured by TOSHIBA Medical Systems Corporation, Tochigi, Japan). The measurement site was the point of greatest masseter muscle bulging during maximal clenching. Measurements were performed under three functional conditions: resting, mouth opening, and clenching. The patient was in the supine position with their head turned sideways, the probe was applied perpendicularly to the skin surface, and the masseter muscle region was selected when the compression and release became constant, and the SVs and cross-sectional area were measured (Figure 1). The SVs and cross-sectional area were measured at resting, mouth opening, and clenching on each side of the left and right masseter muscles at the maximally protruding part of the left and right masseter muscles at maximum clenching. The strain values were obtained using a no-manual-compression method. Therefore, no external pressure was applied during measurement. The strain was derived from subtle vibrations caused by involuntary muscle contractions of the operator’s hand, the patient’s own muscle activity, and respiration. The stability criterion was defined as a low-amplitude and stable strain graph with uniform color distribution on the elastographic image. All measurements were performed twice by the same operator on separate occasions, and the mean value was used for analysis. The intra-operator reliability of the strain value measurements was assessed in 10 randomly selected subjects, yielding an intraclass correlation coefficient (ICC) of 0.92, indicating excellent repeatability.

### 2.3. Factors Related to Masseter Muscle Strain Values

**Figure 2:**
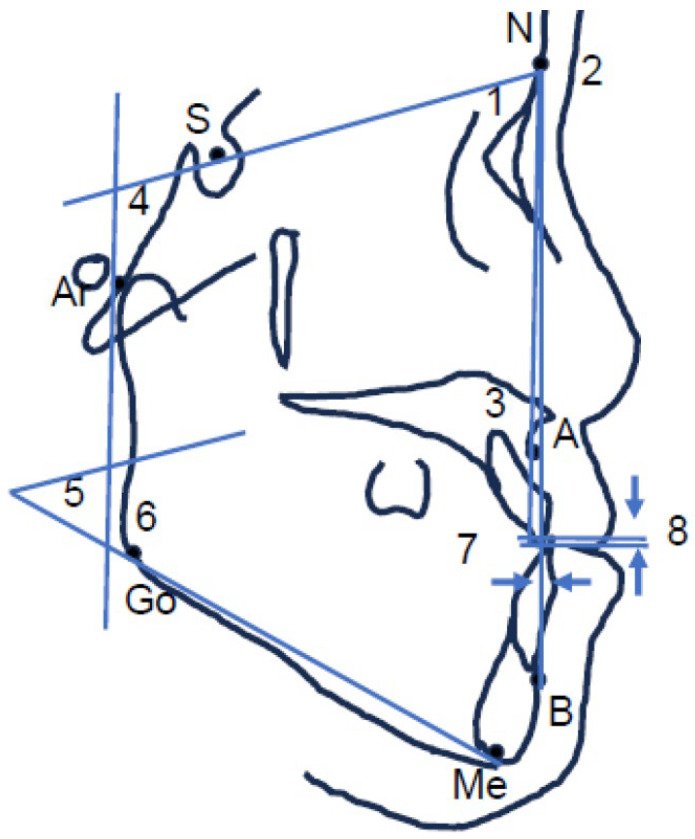
Lateral and frontal cephalometric analysis. Cephalometric landmarks: sella (S), nasion (N), pointA (A), pointB (B), menton (Me), gonion (Go), articulare (Ar), facial midline (FM). Angular measurements: 1. SNAangle; 2. SNB angle; 3. ANB angle; 4. GZN angle; 5. SN-MP angle; 6. Gonial angle. Distance measurements: 7. over jet; 8. over bite. Cephalometric landmarks and reference planes used for skeletal evaluation. Blue lines indicate reference planes (e.g., SN, mandibular plane, and vertical reference lines), and blue arrows represent the direction of horizontal and vertical displacement.

Age, sex, and body mass index were measured as the patient factors. Overjet, overbite, SNA angle, SNB angle, ANB angle, GZN angle, SN-MP angle, and gonial angle by cephalometric analysis were measured as morphological factors (Figure 2). Maximum mouth opening range, masseter muscle area, and SVs at resting, mouth opening, and clenching were measured as oral functional factors.

### 2.4. Statistical Analysis

The SVs of the normal occlusion group and skeletal class II or III dentofacial deformity groups were compared at resting, mouth opening, and clenching. The SVs of dentofacial deformity at resting, mouth opening, and clenching were compared. The cross-sectional areas of the masseter muscle between resting and mouth opening and clenching in patients with dentofacial deformities were compared. The relationship between the functional SVs and the factors was analyzed. For intergroup comparisons, the Kruskal–Wallis test was used, followed by post hoc pairwise comparisons with the Wilcoxon signed-rank test where applicable. Correlations were assessed using linear regression analysis. Statistical analyses were performed using Prism 9 (GraphPad Software, San Diego, CA, USA). A *p* value < 0.05 was considered statistically significant. A post hoc power analysis was conducted using G*Power 3.1 software. Based on the observed effect sizes (Cohen’s d = 0.55) in the comparison of clenching versus resting strain values, the achieved statistical power was 0.83 at α = 0.05, confirming that the sample size (*n* = 86) was adequate for detecting medium effect sizes.

## 3. Results

In total, 60 patients (45 women and 15 men) and 26 volunteers (14 women and 12 men) were included in this study. The average age of the included patients was 26 years (range, 18–59 years), and that of the volunteers was 32 years (range, 23–47 years).

### 3.1. Comparison of Strain Ratio Between Volunteers and Dentofacial Deformities

**Figure 3:**
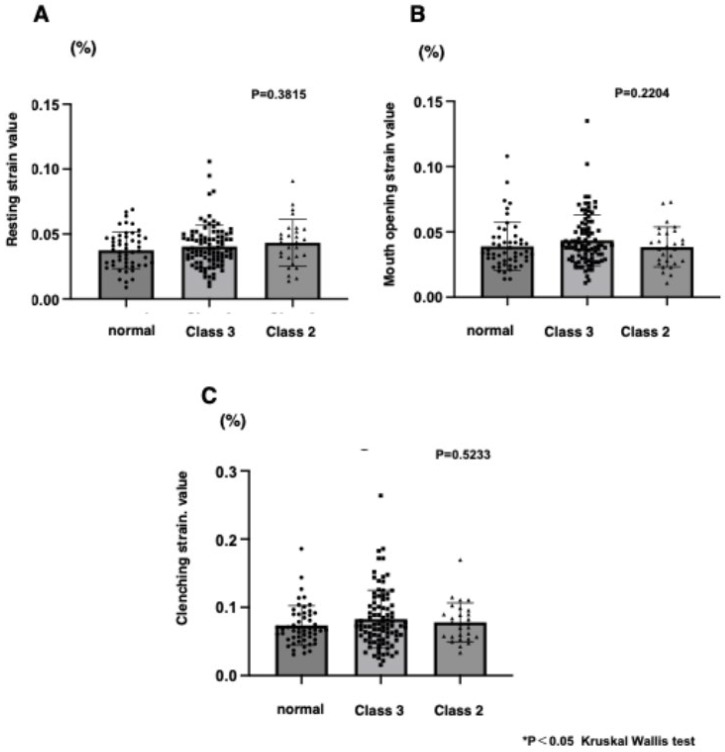
Comparison of masseter muscle strain values among normal and skeletal class III and skeletal class II at resting (**A**), mouth opening (**B**), and clenching (**C**).

**Figure 4:**
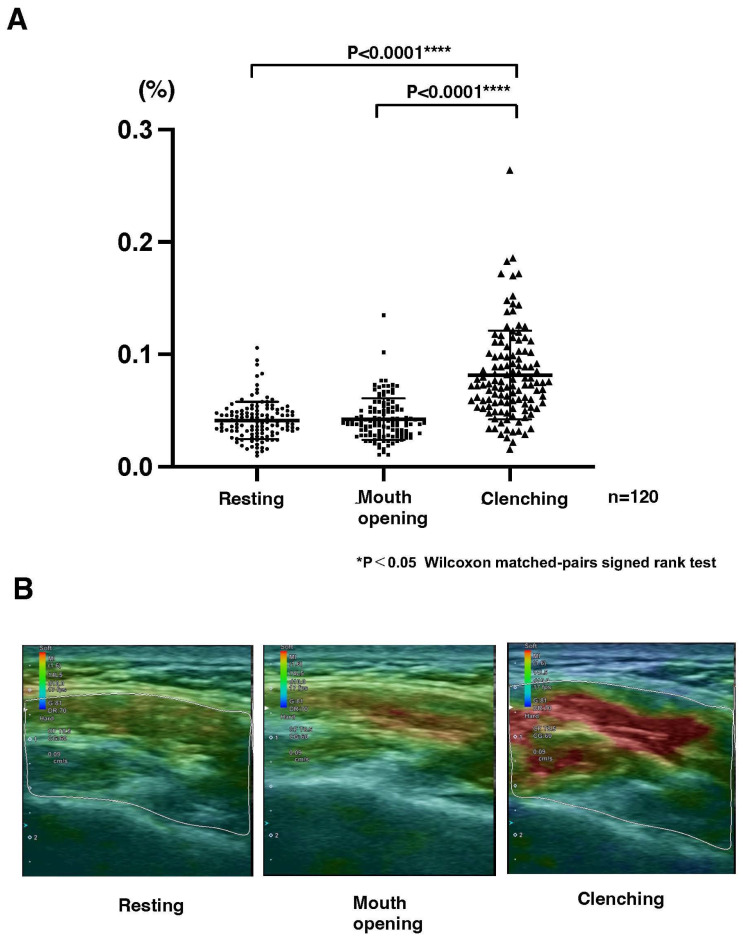
Comparison of masseter muscle strain values among resting, mouth opening, and clenching in patients with dentofacial deformity. (**A**) Strain values of the masseter muscle at rest, during mouth opening, and during clenching. Each dot represents one side of the muscle (*n* = 120). Strain values significantly increased from the resting state to mouth opening and further increased during clenching (**** *p* < 0.0001, Wilcoxon matched-pairs signed rank test) (**B**) Representative strain elastography images of the masseter muscle obtained at rest, during mouth opening, and during clenching. Warmer colors (red) indicate greater strain (softer tissue), whereas cooler colors (blue–green) indicate lower strain (stiffer tissue). The region of interest (ROI) of the masseter muscle is outlined in white.

There were no statistically significant differences among volunteers, skeletal class III, and skeletal class II malocclusion of the SVs at any of the three functional points (Figure 3). Figure 4 shows that SVs at clenching were higher than that at resting (*p* < 0.0001 ****) and at mouth opening (*p* < 0.0001 ****) (A). In the strain elastography images, red represents soft areas, blue represents hard areas, and green represents areas between soft and hard areas. These images show that redness was recognized more prominently during clenching, with an increase in SVs (B).

### 3.2. Comparison of Cross-Sectional Area Among Resting, Mouth Opening, and Clenching in Dentofacial Deformities

**Figure 5:**
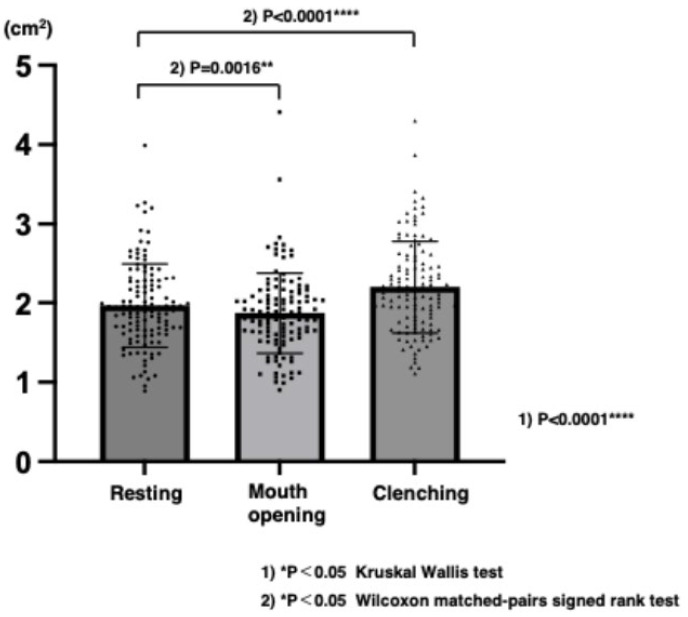
Comparison of masseter muscle cross-sectional area among resting, mouth opening, and clenching in patients with dentofacial deformity.

There was a significant difference in the cross-sectional area of the masseter muscle at resting, mouth opening, and clenching. The cross-sectional area at clenching was the largest and that at mouth opening was the smallest among the three points in time (Figure 5).

### 3.3. Functional Factors Related to Strain Ratio in Dentofacial Deformities

**Figure 6:**
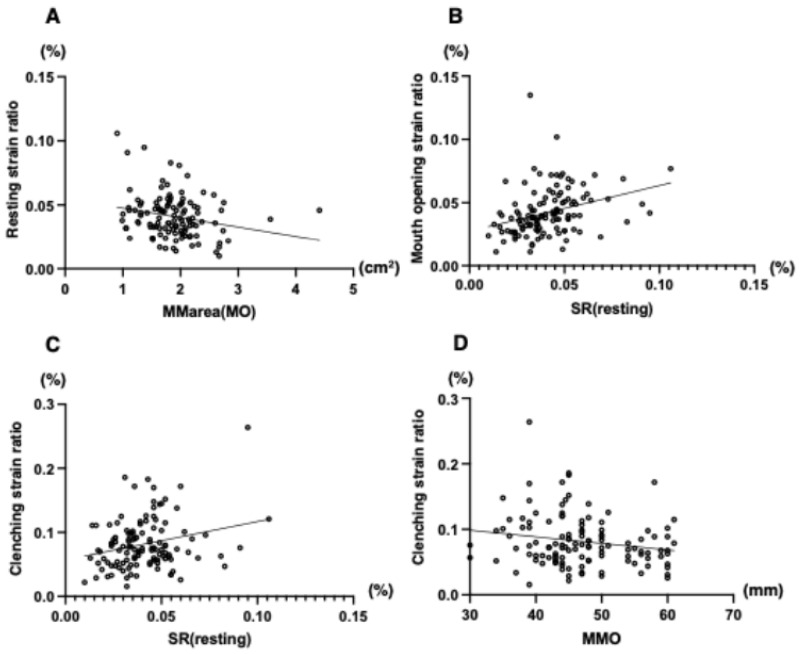
Incidence of masseter muscle strain value at resting (**A**), mouth opening (**B**), and clenching (**C**,**D**) in patients with dentofacial deformity.

Table 1 summarizes the correlations between functional SVs and factors associated with dentofacial deformities. Significant differences were found in the masseter muscle cross-sectional area, the mouth opening SVs and clenching in the resting SVs, the resting SVs with mouth opening, the mouth opening SVs, and the resting SVs with clenching. The resting SVs decreased as the masseter muscle cross-sectional area increased (Figure 6A). The SVs of mouth opening increased as the resting SVs increased (Figure 6B). The SVs of clenching increased as the SVs of resting increased (Figure 6C) and decreased according to mouth opening (Figure 6D).

## 4. Discussion

There were no statistically significant differences among the volunteers, the skeletal class III malocclusion, and the skeletal class II malocclusion of the SVs at any of the three functional points of resting, mouth opening, and clenching. The SVs of the masseter muscle did not change significantly during mouth opening; however, it increased significantly during clenching compared with resting and mouth opening in both dentofacial deformities and normal occlusal volunteers. Although there was no association with anteroposterior maxillofacial skeletal morphology, significant differences were observed during clenching than during resting and mouth opening. We believe that SVs would be useful for masseter muscle functional evaluation because SVs were significantly changed according to functional mandibular movement.

A method for evaluating masticatory muscles is performed using diagnostic imaging methods such as CT, MRI, or electromyogram (EMG) traditionally [11,12,13,14]. Many reports have evaluated the morphology of the masseter muscle in patients with dentofacial deformities using these methods [3,15,16,17], but it is not sufficient to evaluate the stiffness and functional changes in the masseter muscle. Another reason for choosing elastography as an examination method for the masseter muscle is that it can be measured more easily and less invasively than CT, MRI, or EMG, and the characteristics of the muscle during function can be distinguished in real time [7,18]. Ultrasound is a dynamic examination, superior to CT and MRI in lateral resolution and spatial resolution, and is a useful tool for assisting diagnosis. In previous reports, occlusal muscles had a significant difference between resting and clenching in normal occlusion populations, elasticity was increased during maximum contraction [18]. In this study, the SVs significantly increased during clenching, not only in normal occlusion subjects, but also in patients with dentofacial deformities.

Dentofacial deformity is characterized by a decrease in the ratio of type II fibers and atrophy of the masseter muscle fibers [14]. Therefore, we hypothesized that there would be some difference between patients with dentofacial deformities and those with normal occlusion. However, no significant differences were observed between the groups. Although skeletal class II and class III deformities have different biomechanical characteristics, the number of class II patients in this cohort was relatively small, and the subgroup comparison lacked sufficient statistical power. Therefore, both skeletal patterns were merged into one dentofacial deformity group in this study, and this limitation should be considered when interpreting the findings.

After that, we examined the relationship between the masseter muscle cross-sectional area and three points of function; a statistically significant difference was observed during clenching and mouth opening compared to resting. We examined the correlation between the SVs and the cross-sectional area of the masseter muscle, but only the SVs at rest and the cross-sectional area of the masseter muscle when mouth opening was correlated.

This result indicates that the softer the masseter muscle at resting position, the smaller it is at mouth opening. Considering this, the SVs of the masseter muscle at rest may predict mouth opening function.

On the color map image of the masseter muscle, a red area was observed for both mouth opening and clenching. Both contracted and relaxed layers of the masseter muscle were observed at rest and during function. In particular, during clenching, large strains in the masseter muscle (shown in red) were found in the central part, and there was a tendency for the deep layer to have a larger strain than the shallow layer. Differences in muscle layers during function were correlated with the SVs. In a previous ultrasound evaluation of the masseter muscle, the maximum masseter muscle thickness was recorded in the central third of the contracted muscle, and it was greatest in the central region during both contraction and relaxation; it was reported to be thick and thinnest at the bottom [19]. These results are related to the anatomical features of the masseter muscle reported in previous studies. It has been reported that the muscle layer of the masseter muscle has a multi-layered structure, and many studies have shown a layered structure of the masseter muscle [5,20]. High and low strains coexist, and muscle contraction and relaxation differ depending on the layer. A study that analyzed the morphology of the mandible and masseter muscle demonstrated a correlation between the size of the masseter muscle and the size of the processus of the mandibule, and that the more curved the mandibular morphology, the larger the masseter muscle [21,22]. Although morphological factors and SVs in this study showed no correlation, further analysis is recommended after increasing the number of cases.

In the present study, a positive correlation was found between resting SVs and maximum mouth opening, whereas a negative correlation was observed between clenching SVs and maximum mouth opening. These findings suggest that lower resting muscle tension allows greater extensibility of the masseter during mouth opening, resulting in a larger range of motion. Conversely, higher SVs during clenching may reflect increased muscle stiffness or shortening, which could restrict the elongation of the muscle fibers and thereby limit maximum mouth opening. These results indicate that the static and dynamic properties of the masseter muscle may exert opposite influences on mandibular mobility.

Current reports on elastography in the field of maxillofacial and oral surgery include the evaluation of effects before and after treatment for temporomandibular disorders and the evaluation of masseter muscle hardness, thickness, and cross-sectional area before and after surgery [23,24]. However, there have been no reports evaluating changes in the masseter muscle during masticatory muscle function in subjects with jaw deformity and normal occlusion. To our knowledge, this is the first study to demonstrate changes in masseter muscle function during masticatory muscle function in patients with normal occlusion and dentofacial deformities.

Based on the present findings, an ultrasound-based assessment of the masseter SVs may serve as an objective indicator of muscle tension that correlates with mandibular mobility. This technique could be applied for screening patients with excessive muscle tension before orthognathic surgery and for monitoring changes in masticatory muscle stiffness during postoperative rehabilitation. Furthermore, integrating such quantitative muscle assessment into a digital workflow may improve the accuracy of preoperative planning and postoperative follow-up. As demonstrated in the concept of a fully digital workflow for anterior segment repositioning devices [25], digital integration enables real-time functional feedback and patient-specific treatment adjustments. Therefore, combining SV-based muscle evaluation with digital treatment planning could facilitate a more functional approach to the management of jaw deformities.

There are several limitations to this study. First, the sample size was modest, particularly for patients with skeletal class II deformities, resulting in insufficient statistical power for subgroup comparisons. Therefore, class II and III cases were combined into a single dentofacial deformity group, and the results should be interpreted with caution. Second, volumetric or densitometric assessments such as Hounsfield units or grayscale intensity were not included, which may further improve characterization of masseter muscle properties in future studies. Third, the short-term cross-sectional design does not allow evaluation of postoperative adaptation or temporal changes in muscle stiffness. Finally, strain elastography is a semi-quantitative and operator-dependent technique, although standardized probe positioning and repeated average measurements were applied to minimize variability. Future longitudinal studies with larger and more balanced samples are warranted to confirm skeletal pattern-specific differences and to better clarify the functional role of the masseter muscle in dentofacial deformities.

In summary, ultrasound elastography is a simple examination that can evaluate masticatory muscles without radiation exposure and can be expected to provide an objective and more accurate diagnosis. As a result of this study, the SVs measured by ultrasound elastography changed depending on functional mandibular movement and have the potential to evaluate the masseter muscle activity of dentofacial deformities, masseter muscle stiffness, and masseter muscle cross-sectional area during function in patients with dentofacial deformities. Thus, ultrasound elastography may be an effective new tool for evaluating masticatory muscles.

## Figures and Tables

**Table 1:** Relationship between functional strain value and factors.

	Average (Range)	Resting SR		Mouth Opening SR		Clenching SR	
		R^2^	*p* Value	R^2^	*p* Value	R^2^	*p* Value
**(1) Patient factors**							
Age	26 (16–59)	0.03	0.0807	0.02	0.1505	0.01	0.2428
Gender (male/female)	(15/45)	0.01	0.4127	-	0.7436	0.01	0.3326
Body mass index	21.0 (15.8-/31.5)	0.02	0.1601	0.01	0.2698	0.02	0.1209
**(2) Morphological factors**							
Over jet (mm)	−0.4 (−8.5~−12.0)	-	0.9382	0.01	0.2191	0.01	0.3971
Over bite (mm)	0.8 (−7.0~5.0)	-	0.3914	-	0.4405	0.01	0.2683
SNA angle (°)	81.3 (72.3~88.4)	-	0.2078	-	0.7193	-	0.2739
SNB angle (°)	80.9 (69.7~90.3)	0.02	0.1397	-	0.1298	0.02	0.173
ANB angle (°)	0.4 (−5.4~9.6)	0.02	0.1401	-	0.4408	-	0.4759
GZN angle (°)	91.4 (76.2~111.6)	0.02	0.1332	-	0.4434	-	0.5981
SN-MP angle (°)	39.8 (19.6~72.8)	0.03	0.0768	-	0.9733	-	0.4705
Gonial angle (°)	128.5 (107.7~143.7)	-	0.8129	-	0.4627	-	0.8557
**(3) Oral functional factors**							
MMO (mm)	47.3 (34.0–61.0)	0.01	0.0825	-	0.8366	0.03	0.0420 *
MM Area (cm^2^)							
Resting	0.7 (−7.0~5.0)	0.03	0.0825	0.02	0.1726	-	0.2917
Mouth opening	80.0 (73.9~84.8)	0.05	0.0126 *	0.02	0.1451	-	0.397
Clenching	76.6 (67.2~86.7) 0.02	-	0.159	0.01	0.2393	-	0.8172
Strain ratio							
Resting	0.7 (−7.0~5.0)			0.1	0.0003 ***	0.06	0.0055 **
Mouth opening	80.0 (73.9~84.8)	0.1	0.0003 ***			-	0.8142
Clenching	76.6 (67.2~86.7)	0.06	0.0055 **	-	0.8142		

* *p* < 0.05 Linear regression.** *p* < 0.01; *** *p* < 0.001.

## Data Availability

The data supporting the findings of this study are available from the corresponding author upon reasonable request.

## References

[1] Duarte F., Silva J.N., Ramos C., Hopper C. (2024). Anatomic and Functional Masseter Muscle Adaptation Following Orthognathic Surgery: MRI Analysis with Three-Year Follow-Up. Maxillofac. Plast. Reconstr. Surg..

[2] Giannini L., Maspero C., Galbiati G., Kairyte L., Zanoni F., Farronato G. (2017). Orthodontic-Surgical Treatment: Electromyographic and Kinesiographic Evaluation in the Follow-Up Period. Stomatologija.

[3] Kim T.-H., Kim C.-H. (2020). Correlation Between Mandibular Morphology and Masticatory Muscle Thickness in Normal Occlusion and Mandibular Prognathism. J. Korean Assoc. Oral Maxillofac. Surg..

[4] Nakayama M., Ariji Y., Nishiyama W., Ariji E. (2015). Evaluation of Masseter Muscle Elasticity Using Acoustic Coupling Agents as References in Strain Sonoelastography. Dentomaxillofac. Radiol..

[5] Etöz M., Demirbaş A.E., Topsakal K.G., Etöz O.A., Kaya M., Alkan A. (2020). Sonoelastographic Evaluation of the Masseter Muscle Before and After Mandibular Setback Surgery. Niger. J. Clin. Pract..

[6] Winn N., Lalam R., Cassar-Pullicino V. (2016). Sonoelastography in the Musculoskeletal System: Current Role and Future Directions. World J. Radiol..

[7] Ashir A., Jerban S., Barrère V., Wu Y., Shah S.B., Andre M.P., Chang E.Y. (2023). Skeletal Muscle Assessment Using Quantitative Ultrasound: A Narrative Review. Sensors.

[8] Coclici A., Hedeşiu M., Bran S., Băciuţ M., Dinu C., Rotaru H., Roman R. (2019). Early and Long-Term Changes in the Muscles of the Mandible Following Orthognathic Surgery. Clin. Oral Investig..

[9] Ueki K., Marukawa K., Hashiba Y., Nakagawa K., Degerliyurt K., Yamamoto E. (2008). Relationship Between Recovery of Maximum Mandibular Opening and Maxillomandibular Fixation Period After Orthognathic Surgery. J. Oral Maxillofac. Surg..

[10] Lee D.H., Yu H.S. (2012). Masseter Muscle Changes Following Orthognathic Surgery: A Long-Term Three-Dimensional CT Follow-Up. Angle Orthod..

[11] Olchowy C., Więckiewicz M., Sconfienza L.M., Łasecki M., Seweryn P., Smardz J., Hnitecka S., Dominiak M., Olchowy A. (2020). Potential of Using Shear Wave Elastography in the Clinical Evaluation and Monitoring of Changes in Masseter Muscle Stiffness. Pain Res. Manag..

[12] Olchowy A., Więckiewicz M., Malysa A., Olchowy C. (2021). Determination of Reference Values of the Masseter Muscle Stiffness in Healthy Adults Using Shear Wave Elastography. Int. J. Environ. Res. Public Health.

[13] Takashima M., Arai Y., Kawamura A., Hayashi T. (2017). Quantitative Evaluation of Masseter Muscle Stiffness in Patients with Temporomandibular Disorders Using Shear Wave Elastography. J. Prosthodont. Res..

[14] Sciote J.J., Raoul G., Ferri J., Close J., Horton M.J., Rowlerson A. (2013). Masseter Function and Skeletal Malocclusion. Rev. Stomatol. Chir. Maxillofac. Chir. Orale.

[15] Impellizzeri A., Serritella E., Putrino A., Vizzielli G., Polimeni A., Galluccio G. (2019). Assessment of Masticatory and Cervical Muscle Thickness by Ultrasonography in Patients with Facial Asymmetry. Clin. Ter..

[16] Yamada T., Sugiyama G., Mori Y. (2020). Masticatory Muscle Function Affects the Pathological Conditions of Dentofacial Deformities. Jpn. Dent. Sci. Rev..

[17] Ueki K., Takazakura D., Marukawa K., Shimada M., Nakagawa K., Yamamoto E. (2006). Relationship Between the Morphologies of the Masseter Muscle and the Ramus and the Occlusal Force in Patients with Mandibular Prognathism. J. Oral Maxillofac. Surg..

[18] Durão A.P.R., Morosolli A., Brown J., Jacobs R. (2017). Masseter Muscle Measurement Performed by Ultrasound: A Systematic Review. Dentomaxillofac. Radiol..

[19] Gawriołek K., Klatkiewicz T., Przystańska A., Maciejewska-Szaniec Z., Gedrange T., Czajka-Jakubowska A. (2021). Standardization of the Ultrasound Examination of the Masseter Muscle with Size-Independent Calculation of Records. Adv. Clin. Exp. Med..

[20] Akita K., Fukino K. (2022). The Significance and Classification of the Layered Structures of the Human Masseter and Temporalis. Ann. Anat..

[21] Sella-Tunis T., Pokhojaev A., Sarig R., O’Higgins P., May H. (2018). Human Mandibular Shape Is Associated with Masticatory Muscle Force. Sci. Rep..

[22] Pepicelli A., Woods M., Briggs C. (2005). The Mandibular Muscles and Their Importance in Orthodontics: A Contemporary Review. Am. J. Orthod. Dentofac. Orthop..

[23] Taşdemir Z., Etöz M., Köy Ö., Soydan D., Alkan A. (2020). Masseter Muscle Thickness and Elasticity in Periodontitis. J. Oral Sci..

[24] Lee K., Chon S. (2021). Assessment of Masseter and Sternocleidomastoid Muscle Thickness and Tonicity and Maximum Mouth Opening in Patients with Temporomandibular Disorder. Healthcare.

[25] Tra N.T., Anh N.V., Duc N.M. (2025). A completely digital workflow for an anterior repositioning device. J. Prosthet. Dent..

